# Experimental Design-Based Response Surface Methodology Optimization for Synthesis of *β*-Mercapto Carbonyl Derivatives as Antimycobacterial Drugs Catalyzed by Calcium Pyrophosphate

**DOI:** 10.1155/2014/586437

**Published:** 2014-03-06

**Authors:** Younes Abrouki, Abdelkader Anouzla, Hayat Loukili, Jamal Bennazha, Rabiaâ Lotfi, Ahmed Rayadh, My Abdellah Bahlaoui, Saïd Sebti, Driss Zakarya, Mohamed Zahouily

**Affiliations:** ^1^Department of Research, Faculty of Science and Technology, University Hassan II, Mohammedia 20652, Morocco; ^2^Department of Chemistry, Faculty of Science Ben M'Sik, University Hassan II, Casablanca 20455, Morocco

## Abstract

A simple protocol for the efficient preparation of *β*-mercapto carbonyl derivatives as antimycobacterial drugs has been achieved via Thia-Michael reaction between chalcones derivatives and thiols in the presence of calcium pyrophosphate as a heterogeneous catalyst under mild reaction conditions. The central composite design was used to design an experimental program to provide data to model the effects of various factors on reaction yield (*Y*). The variables chosen were catalyst weight (*X*_1_), reaction time (*X*_2_), and solvent volume (*X*_3_). The mathematical relationship of reaction yield on the three significant independent variables can be approximated by a nonlinear polynomial model. Predicted values were found to be in good agreement with experimental values. The optimum reaction conditions for reaction model (chalcone and thiophenol) obtained by response surface were applied to other substrates. This procedure provides several advantages such as high yield, clean product formation, and short reaction time.

## 1. Introduction

Tuberculosis is the second most common cause of death from infectious disease. Roughly one-third of the world's population has been infected with* Mycobacterium tuberculosis*, and new infections occur at a rate of one per second. In 2007 there were an estimated 13.7 million chronic active cases, and in 2010 there were 8.8 million new cases and 1.45 million deaths. 0.35 million of these deaths occur in those coinfected with HIV [1].

The needs of newly developed antimycobacterial drugs are required for the control of tuberculosis in the present time. In the discovery of new antimycobacterial drugs, the emergence of multidrug-resistant and extensively drug-resistant strains of* Mycobacterium* has encouraged the researchers to intensify the efforts to discover novel drugs [2].

Recently, the Czech authors showed that the 1,3-diphenyl-3-arylsulfenylpropan-1-one derivatives presented an antimycobacterial activity [3].

Thia-Michael reaction is a convenient route for synthesis of these *β*-mercapto carbonyl derivatives [4]. Traditionally, this reaction is catalyzed by strong bases [5] such as alkali metal alkoxides, hydroxides, and amines. However the use of either strongly acidic or basic conditions frequently leads to the formation of undesirable side products owing to competing reactions, such as polymerization, self-condensation, and rearrangements. The development of solid basic catalysts which could replace the liquid bases currently used in industrial processes is a major field of today's catalysis research [6].

In this paper we report a mild and convenient method for synthesis of *β*-mercapto carbonyl derivatives as antimycobacterial drugs catalyzed by calcium pyrophosphate using central composite experimental design [7].

## 2. Materials and Methods

### 2.1. Preparation and Characterization of Catalyst

The calcium pyrophosphate (CP) was prepared by the dehydration of CaHPO_4_ at 750°C for 2 h. The powder was dried for 2 hours at 100°C before use in order to eliminate possible water molecules adsorbed on the surface of the sample which may affect catalytic activity [8]. Analysis of CP catalyst by X-ray diffraction showed a single phase of *β*-Ca_2_P_2_O_7_. The analysis of morphology for this catalyst by scanning electron micrograph showed that the particle size of CP powder is less than 1 *μ*m.

### 2.2. General Procedure

**Figure 1:**
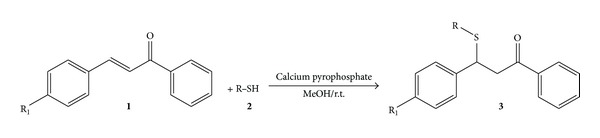
Synthesis of *β*-mercapto carbonyl derivatives.

The general procedure for synthesis of *β*-mercapto carbonyl derivatives is reported in Figure 1 as follows: to a flask containing an equimolar mixture (1 mmol) of chalcones, derivatives as Michael acceptors** 1** and thiols as Michael donors** 2** in methanol catalyzed by CP were added, and the mixture was stirred at room temperature until completion of the reaction, as monitored by thin layer chromatography.

The catalyst was filtered and washed with dichloromethane, and the filtrate was concentrated under reduced pressure. The crude product was purified by crystallization. The product was analyzed by ^1^H, ^13^C NMR, and IR spectrometry.

*1,3-Diphenyl-3-phenylsulfenylpropan-1-one (3a).* White solid, mp 116–118°C; Rf (10% AcOEt/hexane) 0.36; *υ*max (KBr)1680 cm^−1^;*δ*H (400 MHz CDCl3) 3.5 (H, CH2, dd, *J*1 = 6.2 Hz, *J*2 = 17.2 Hz); 3.7 (H, CH2, dd, *J*1 = 8.1 Hz, *J*2 = 17.2 Hz); 4.98 (H, CH, t, *J* = 7.2 Hz); 7.2–7.6 (13H, arom, m); 7.9 (2H, arom, d, *J* = 7.5); *δ*C (100 MHz CDCl3) 44.7; 48.3; 127.4; 127.6; 127.9; 128.1; 128.5; 128.6; 128.9; 132.8; 133.3; 134.6; 136.8; 141.3; 197.9; MS (*m/z*): 318 (M+); 206; 109. 

*3-(4-Chlorophenyl)-1-phenyl-3-phenylsulfenylpropan-1-one (3b).* White solid; mp 64–67°C; Rf (10% AcOEt/hexane) 0.35; *υ*max (KBr) 1730  cm^−1^; *δ*H (400 MHz CDCl3) 3.6 (2H, CH2, m); 4.97 (H, CH, t, *J* = 7.2 Hz); 7.2–7.5 (12H, arom, m); 7.9 (2H, arom, d, *J* = 7.5 Hz); *δ*C (100 MHz CDCl3) 44.2; 47.8; 122.3; 122.7; 128.1; 128.3; 128.8; 129.1; 129.3; 132.9; 133.4; 133.6; 134.2; 136.3; 143.8; 148.2; 196.1; MS (*m/z*): 353 (M+); 241.91; 109. 

*3-(4-Methoxyphenyl)-1-phenyl-3-phenylsulfenyl-propan-1-one (3c).* White solid; 83–85°C; Rf (10% AcOEt/hexane) 0.26; IR (KBr, *ν* cm^−1^): 1730  cm^−1^; 1H NMR (400 MHz, CDCl3) *δ*: 3.62 (2H, dd, *J*1 = 7.5 Hz, *J*2 = 15.2 Hz, *J*3 = 17.2 Hz, CH2), 3.76 (3H, s, OCH3); 4,92 (H, dd, *J*1 = 6,2 Hz, *J*2 = 8.1 Hz, CH), 6.78 (2H, m, *J* = 8.6 Hz, arom), 7.1–8.05 (12H, m, arom); 13C NMR (100 MHz, CDCl3) *δ*: 197.19, 158.77, 144.7, 136.78, 134.48, 133.13, 129.1, 128.63, 128.09, 127.47, 119.82, 114.46, 113.86, 55.33, 47.67, 44.86; MS (*m/z*): 348 (M+), 239, 238, 237, 109. 

*3-(2-Aminophenylsulfenyl)-1,3-diphenylpropan-1-one (3d).* White solid; mp 126–128°C; Rf (10% AcOEt/hexane) 0.20; *υ*max (KBr) 1710; 3410  cm^−1^; *δ*H (400 MHz CDCl3) 3.5 (H, CH2, dd, *J*1 = 7.0 Hz, *J*2 = 17.4 Hz); 3.7 (H, CH2, dd, *J*1 = 7.3 Hz, *J*2 = 17.4 Hz); 4.3 (2H, NH2, bs); 4,73 (H, CH, t, *J* = 7.1 Hz); 6.53–7.54 (12H, arom, m); 7.9 (2H, arom, d, *J* = 7.2 Hz); *δ*C (100 MHz, CDCl3) 44.1; 47.1; 114.9; 115.3; 115.7; 118.0; 118.2; 127.3; 127.6; 128.1; 128.4; 128.6; 130.6; 131.6; 133.3; 136.8; 137.7; 141.7; 148.7; 149.5; 197.2; MS (*m/z*): 333 (M+); 207; 126. 

*3-(2-Aminophenylsulfenyl)-3-(4-chlorophenyl)-1-phenylpropan-1-one (3e).* White solid; mp 78–81°C; Rf (10% AcOEt/hexane) 0.33; *υ*max (KBr) 1700; 3470  cm^−1^; *δ*H (400 MHz CDCl3) 3.4 (H, CH2, dd, *J*1 = 7.1 Hz, *J*2 = 17.5 Hz); 3.6 (H, CH2, dd, *J*1 = 7.2 Hz, *J*2 = 17.5 Hz); 4.1 (2H, NH2, bs); 4.9 (1H, CH, t, *J* = 7.1 Hz); 6.5–7,5 (11H, arom, m); 7.9 (2H, arom, d, *J* = 7.2 Hz); *δ*C (100 MHz CDCl3) 43.4; 45.8; 115.2; 118.3; 118.8; 122.2; 122.5; 128.1; 128.7; 129.1; 131.1; 131.6; 133.6; 134.1; 136.8; 137.5; 148.6; 149.4; 196. MS (*m/z*) 367 (M+); 242; 111. 

*3-(2-Aminophenylsulfenyl)-3-(4-methoxyphenyl)-1-phenyl-propan-1-one (3f).* White solid; 108–111°C; Rf: (10% AcOEt/hexane) 0.24; IR (KBr, *ν* cm^−1^)*: *3420, 1730  cm^−1^; 1H NMR (400 MHz, CDCl3) *δ*: 3.52 (2H, dd, *J*1 = 7.5 Hz, *J*2 = 15.2 Hz, *J*3 = 17.3 Hz, CH2), 3.76 (3H, s, OCH3), 4.2 (2H, s, NH2), 4,8 (H, dd, *J*1 = 6,2 Hz, *J*2 = 8.1 Hz, CH), 6.48–7.76 (11H, m, arom), 7.95 (2H, d, *J* = 7.4 Hz, arom); 13C NMR (100 MHz, CDCl3) *δ*: 19736, 158.74, 149.5, 137.69, 136.33, 135.10, 133.25, 131.13, 130.62, 129.71, 129.72, 128.10, 127.49, 127.19, 125.32, 123.01, 118.07, 114.88, 114.09, 113.77, 55.30, 46.32, 44.32; MS (*m/z*): 363 (M+), 239, 238, 237, 125.

### 2.3. Statistical Analysis

A central composite rotatable design for *k* independent variables was employed to design the experiments in which the variance of the predicted response, *Y*, at some points of independent variables, *X*, is only a function of the distance from the point to the design centre.

The design of experiment is intended to reduce the number of experiments and to arrange the experiments with various combinations of independent variables. In the rotatable design, the standard error, which depends on the coordinates of the point on the response surface at which Ŷ is evaluated and on the coefficients *β*, is the same for all points that have the same distance from the central point.

These designs consist of a 2^*k*^ factorial (coded to the usual ±1 notation) augmented by 2∗*k* axial points (±*α*, 0, 0), (0, ±*α*, 0), (0, 0, ±*α*) and 2 centre points (0, 0, 0). The value of *α* for rotatability depends on the number of points in the factorial portion of the design, which is given in

(1) $$\begin{array}{c} \alpha ={\left(N_{F}\right)}^{1/4}, \end{array}$$

where *N*_*F*_ is the number of points in the cube portion of the design (*N*_*F*_ = 2^*k*^, *k* is the number of factors). Since there are three factors, the *N*_*F*_ number is equal to 8 points, while *α* is equal to 1.682 according to (1). In this study, the response was reaction yield. Each response was used to develop an empirical model that correlated the response to the reaction conditions variables for synthesis of an antimycobacterial drug, using a second-degree polynomial equation as given by

(2) $$\begin{array}{c} \text{Ŷ}=\beta_{0}+\Sigma \beta_{i}X_{i}+\Sigma \beta_{ii}{{\text{X}}_{\text{i}}}^{2}+\Sigma \beta_{ij}X_{i}X_{j}, \end{array}$$

where *β*_0_ is the constant coefficient, *β*_*i*_ is the linear coefficients, *β*_*ij*_ is the cross-product coefficients, and *β*_*ii*_ is the quadratic coefficients. The software Statgraphics-Plus was used for the experimental design, data analysis, model building, and graph plotting.

## 3. Results and Discussion

Conjugate addition between chalcone (R_1_=H) and thiophenol (R=Ph) was chosen as model substrates to determine suitable reaction conditions for synthesis of 1,3-diphenyl-3-phenylsulfenylpropan-1-one catalyzed by CP in methanol at room temperature.

Preliminary experiments were carried out to screen the appropriate parameters and to determine the experimental domain. From these experiments, the effects of catalyst weight (*X*_1_), reaction time (*X*_2_), and solvent volume (*X*_3_) are investigated on reaction yield as response. The parameter levels and coded values were given in Table 1.

The experimental design matrix and the corresponding experimental parameters and response value were shown in Table 2.

The 10 coefficients of this design are easily calculated by the least squares method. The significance of effects can be estimated by comparing the *F* distribution of the experimental values (*F*_exp⁡_) to a critical value (*F*_0.05_(1.6) = 5.99). According to the results showed in Table 3.

In this case, the linear terms (*X*_1_, *X*_2_, and *X*_3_) and the squared terms (*X*_1_^2^, *X*_2_^2^, and *X*_3_^2^) were significant model terms whereas the interaction terms (*X*_1_ *X*_2_, *X*_1_ *X*_3_, and *X*_2_ *X*_3_) were insignificant to the response. The final empirical model in terms of coded factors after excluding the insignificant terms for reaction yield is shown in

(3) Y=93.9167+7.7982∗X1+9.023∗X2+4.5565∗X3−6.6629∗X12−7.7235∗X22−4.188∗X32.

Positive sign in front of the terms indicates synergistic effect, whereas negative sign indicates antagonistic effect.

The analysis of variance (ANOVA) for model of the reaction yield is listed in Table 4. From this ANOVA, the value of the Model *F*_exp⁡_ was 13.6956 (>F_0.01_(9.6) = 7.98) implying that the model was significant.

The quality of the model developed was evaluated based on the correlation coefficient value (*R*). The *R* value for (3) was 0.9765. The *R* value obtained was relatively high, indicating that there was a good agreement between the experimental and the predicted values from the model.

The *R* ^2^ value for (3) was 0.9536. This indicated that 95.36% of the total variation in the reaction yield was attributed to the experimental variables studied. From the statistical results obtained, it was shown that the above model was adequate to predict the reaction yield within the range of variables studied.

The investigation of (3) showed that, if *X*_1_ = 0.5, *X*_2_ = 0, and *X*_3_ = −0.25, the value predicted from the results using response surface model is 95%. The experimental checking in this point, that is, under the optimum reaction conditions such as catalyst weight = 160 mg, reaction time = 30 min, and solvent volume = 1.75 mL with high reaction yield 94% of product** 3a,** confirms this result.

To determine the scope and limitation for synthesis of *β*-mercapto carbonyl derivatives, the optimum reaction conditions of product** 3a** were applied to other substrates as shown in Table 5.

In this study, the Thia-Michael reaction between several chalcones and thiols has been used for synthesis of *β*-mercapto carbonyl derivatives.

The yields obtained of *β*-mercapto carbonyl derivatives are very high and exceed 92% in short reaction time, except for product (**3c**). The products of undesirable side reactions resulting from 1, 2 addition, polymerization, and bis-addition are not observed. The use of CP catalyst is particularly interesting since it is regenerated by calcinations at 750°C during 15 min, and after five successive recoveries, sulfanyl products were obtained with same yield.

## 4. Conclusion

In conclusion, the optimization of reaction conditions for synthesis of *β*-mercapto carbonyl derivatives catalyzed by calcium pyrophosphate has been studied using central composite design. This work-up procedure provides a clean, fast, high yielded, environmentally friendly, and simple route to synthesis of antimycobacterial drugs.

## Figures and Tables

**Table 1:** Study field and coded factors.

Natural variable	Unit	Coded variables *X*_1_, *X*_2_, and *X*_3_		
		−1	0	+1
Catalyst weight	mg	40	120	200
Reaction time	min	20	30	40
Solvent volume	mL	1	2	3

**Table 2:** Experimental design and results.

Order	Coded units of variables			Reaction yield
	*X*_1_	*X*_2_	*X*_3_	
01	+1	+1	+1	96
02	+1	+1	−1	82
03	+1	−1	+1	74
04	+1	−1	−1	68
05	−1	+1	+1	78
06	−1	+1	−1	69
07	−1	−1	+1	65
08	−1	−1	−1	57
09	−1.682	0	0	61
10	+1.682	0	0	94
11	0	−1.682	0	56
12	0	+1.682	0	93
13	0	0	−1.682	77
14	0	0	+1.682	92
15	0	0	0	94
16	0	0	0	93

**Table 3:** Estimated coefficients of the model and their significances.

Source of variation	Coefficient	Sum of squares	*ν*	Mean square	*F*_exp⁡_	Significance test
*β*_0_	93.9167	—	1	—	—	
*β*_1_	07.7982	0830.504	1	0830.504	34.87	∗
*β*_2_	09.0230	1111.880	1	1111.880	46.68	∗
*β*_3_	04.5565	0283.534	1	0283.534	11.90	∗
*β*_11_	−06.6629	0411.269	1	0411.269	17.27	∗
*β*_12_	01.3750	0015.125	1	0015.125	00.63	NS
*β*_13_	00.3750	0001.125	1	0001.125	00.05	NS
*β*_22_	−07.7235	0552.631	1	0552.631	23.20	∗
*β*_23_	01.1250	0010.125	1	0010.125	00.43	NS
*β*_33_	−04.1880	0162.485	1	0162.485	06.82	∗

*Significant at a level of 5%; NS: not significant.

**Table 4:** Regression variance analysis for the model.

Source of variation	Sum of squares	*ν*	Mean square	*F*_exp⁡_	Significance test
Regression	2936.022	9	326.2246	13.6956	∗
Residue	142.918	6	23.8196	—	—

Total	3078.94	15	—	—	—

*Significant at a level of 1%; NS: not significant.

**Table 5:** Synthesis of sulfanyl derivatives.

Products	R_1_	R	Yield %
**3a**	H	–Ph	94
**3b**	Cl	–Ph	96
**3c**	OMe	–Ph	77
**3d**	H	–2-NH_2_-Ph	93
**3e**	Cl	–2-NH_2_-Ph	95
**3f**	OMe	–2-NH_2_-Ph	92
